# Impact of the ‘10,000 lives’ program on Quitline referrals, use and outcomes by demography and Indigenous status

**DOI:** 10.1111/dar.13499

**Published:** 2022-07-13

**Authors:** Arifuzzaman Khan, Kalie Green, Linda Medlin, Gulam Khandaker, Sheleigh Lawler, Coral Gartner

**Affiliations:** ^1^ School of Public Health The University of Queensland Brisbane Australia; ^2^ Central Queensland Public Health Unit Central Queensland Hospital and Health Service Rockhampton Australia; ^3^ Aboriginal and Torres Strait Islander Health and Wellbeing Central Queensland Hospital and Health Service Rockhampton Australia

**Keywords:** impact evaluation, quitline, regional initiative, smoking cessation, sub‐group analysis

## Abstract

**Introduction:**

In November 2017, Central Queensland Public Health Unit launched the ‘10,000 Lives’ initiative to reduce smoking prevalence in Central Queensland. The program partnered with local champions and other programs (e.g. ‘Deadly Choices’) to promote the use of smoking cessation services (e.g. Quitline) in Central Queensland. This study assesses the program's impact on Quitline use by participant demographics and Indigenous status.

**Methods:**

We compared the number of referred individuals who participated in and completed the Quitline program, and quit smoking during 26‐months before (July 2015 to August 2017) and after (November 2017 to December 2019) the ‘10,000 Lives’ launch. We conducted an interrupted time series analysis of monthly referrals to and use of Quitline for Aboriginal and Torres Strait Islander peoples.

**Results:**

Overall, 3207 individuals were referred to Quitline during the 26‐months‐post‐launch compared to 1594 during 26‐months‐pre‐launch period of ‘10,000 Lives’. The number of referred individuals who completed Quitline program increased by 330.7% and quit smoking by 308.3% in post‐launch period. The increase was substantially higher among aged 45+ years, females and Aboriginal and Torres Strait Islander peoples. The result for referrals and use of Quitline was validated by interrupted time series analysis for Aboriginal and Torres Strait Islander peoples.

**Discussion and Conclusions:**

The ‘10,000 Lives’ collaborative approach to partner with local champions and targeted smoking cessation programs was effective in increasing the use of Quitline and smoking cessation among all demographic groups, including Aboriginal and Torres Strait Islander peoples. This approach can be used in other regions to address higher smoking prevalence.

## 1 INTRODUCTION

Smoking remains a leading preventable cause of premature death globally [1]. In Australia, smoking was responsible for 8.6% of the total disease burden in 2018 [2]. Smoking has declined in recent decades, but rates vary by gender, age and ethnicity [3, 4]. In Central Queensland (CQ), a regional district with a population of around 220,000, 15.4% of adults smoked daily in 2020, compared to 12.9% nationally [5]. Smoking was highest among males (18.9%), persons aged 45–64 years (18.5%) and people living in the most disadvantaged areas (24.6%) [5]. In particular, reducing smoking among Aboriginal and Torres Strait Islander peoples is a priority as it is estimated to cause one‐third of all deaths among this population [6]. Smoking is declining among Aboriginal and Torres Strait Islander peoples, yet remains high due to the ongoing impacts of colonisation, with 42% of adults smoking daily in 2018–2019 [5].

A range of smoking cessation support services are available in Australia, including Quitline since 1997 [7]. Queensland Health tailors it's Quitline service to different population groups [8], and introduced an Intensive Quit Support Program (IQSP) in 2005, comprised of counselling sessions and 12 weeks of free nicotine replacement therapy (NRT) mailed to clients [9]. The IQSP was initially offered to Queensland Health staff when smoking was banned at all Queensland Health facilities, but was expanded in 2011 to include blue‐collar workers participating in a Workplace Quit Smoking Program [9]. Additional groups have been added over time, including regional and remote areas with a smoking prevalence greater than 14% (including CQ) [9]. Queensland Health also runs health promotion campaigns for the general population and priority population groups, including pregnant women and Aboriginal and Torres Strait Islander peoples (e.g. ‘My smoking Quitline’, ‘all by myself’, ‘Quit for You…Quit for Baby’, ‘Quit for You’ and ‘Yarn to Quit’) [10].

To maximise the impact of these smoking cessation activities in CQ, the local Public Health Unit launched a coordinated campaign named ‘10,000 Lives’ in November 2017 [11]. The key strategy was identifying and encouraging champions from clinical and community services to refer smoking clients or colleagues to Quitline. The ‘10,000 Lives’ strategy was adapted from the previously successful ‘10,000 Steps Rockhampton’ program, which promoted physical activity within the same region [12, 13]. To increase referrals to Quitline for Aboriginal and Torres Strait Islander peoples, ‘10,000 Lives’ collaborated with existing culturally appropriate programs, including Deadly Choices, a health promotion initiative for Aboriginal and Torres Strait Islander peoples [14], and B.strong, a training program for delivering brief interventions to Aboriginal and Torres Strait Islander peoples [15]. The ‘10,000 Lives’ initiative also encouraged Queensland Health staff in the oral health, mental health and antenatal health units to participate in a Quality Improvement Payment program, which provided incentives to their unit for meeting completion targets for the Smoking Cessation Clinical Pathway. A detailed description of the ‘10,000 Lives’ program has been reported elsewhere [16].

We previously demonstrated that compared to other regional areas of Queensland without a comparable program, the introduction of ‘10,000 Lives’ accounted for an increase in the rate of monthly referrals to, and smoking cessation counselling sessions completed with Quitline in CQ overall [17]. However, that analysis did not examine whether priority population groups (e.g. males, 45–64 years age group and Aboriginal and Torres Strait Islander peoples) similarly benefitted. This study evaluated the impact of ‘10,000 Lives’ by demographic characteristics, including Indigenous status.

## 2 METHODS

We aimed to compare the number of referrals to, and use of Quitline services, and the number who successfully quit smoking before and after the launch of ‘10,000 Lives’ by demographic characteristics (sex and age) and Indigenous status. We also aimed to determine the impact of the program on monthly rates of referral to and use of Quitline for Aboriginal and Torres Strait Islander peoples.

### 2.1 Data access

We accessed Quitline data from Queensland's Health Contact Centre for 2014–2019. Two datasets were accessed; dataset 1: individual‐level Quitline data for all clients, and dataset 2: monthly counts of referrals to, participation in (initial counselling session completed) and interaction with (total counselling sessions completed) Quitline for Aboriginal and Torres Strait Islander peoples. We used dataset 1 to explore the impact of ‘10,000 Lives’ by demographic characteristics and Indigenous status. The impact was estimated as changes in the number of referrals to and use of Quitline, and smoking cessation outcomes, in the post‐launch period. An interrupted time series (ITS) analysis of dataset 2 evaluated the impact of ‘10,000 Lives’ solely for Aboriginal and Torres Strait Islander peoples. The datasets were analysed in R [18]. We excluded a two‐month buffer period (1 September 2017 to 31 October 2017) to allow a comparison of Quitline data prior to the ‘10,000 Lives’ program and after the program was fully operational.

### 2.2 Quitline referrals and use, and smoking cessation outcomes: Analysis of individual level Quitline data

**FIGURE 1:**
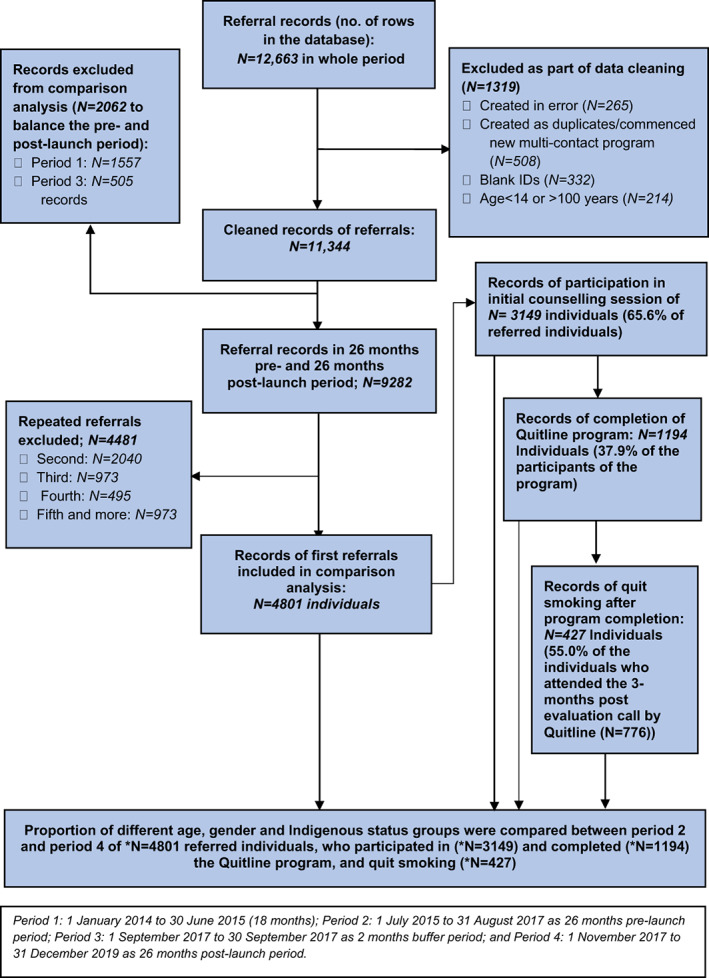
Flow diagram of analysis of individual level Quitline data

Dataset 1 (anonymised individual level data for referred individuals) contained 12,663 records of referrals to Quitline from 1 January 2014 to 31 December 2019, including age, gender, Indigenous status (Aboriginal and/or Torres Strait Islander, or neither), referral source, Quitline program participation, completion and outcome (smoking status). The period was initially divided into four: period 1 (1 January 2014 to 30 June 2015 = 18 months); period 2 (1 July 2015 to 31 August 2017 = 26‐month pre‐launch period); period 3 (1 September 2017 to 31 October 2017 = two‐month buffer); and period 4 (1 November 2017 to 31 December 2019 = 26‐month post‐launch period). After data cleaning (removing erroneous/duplicate/incomplete records), the dataset contained 11,444. We included only period 2 (26 months) and period 4 (26 months) in our comparative statistical analysis to balance the pre‐ and post‐launch periods. We excluded the repeat referrals (second *n* = 2040, third *n* = 973, fourth *n* = 495 and fifth or more *n* = 973) from the analysis. So, we analysed the records of *n* = 4801 referred individuals in our analysis. Multiple outcome measures were included in this analysis. These were the demographic distribution (i.e. age, gender) and Indigenous status of the referred individuals who: (i) *participated* in the program by attending the initial counselling session with a Quitline counsellor including an assessment and quit support plan (*n* = 3149); (ii) *completed* the program by attending four counselling sessions with the Quitline counsellor (*n* = 1194); and (iii) *smoking cessation outcome* (*n* = 427) and the change in these numbers in the 26‐month‐post‐launch period when compared to the 26‐month‐pre‐launch period. Smoking cessation was defined as abstinent from smoking for at least 3 months from program completion to the program evaluation call. Abstinence from smoking was determined based on the responses to the Quitline counsellor's question to the participant during evaluation call: *‘Are you currently smoking? If not, how long did you quit for?*’. We additionally explored: referral source of the referred individuals; and any quit assistance used (e.g. NRT) for the individuals who attended the evaluation call made by Quitline. Figure 1 shows the development of dataset 1. We performed chi‐square tests to compare the proportions of each categorical variable and analysis of variance for continuous variables. We calculated the relative change (%) in the numbers of each outcome in the post‐launch period in comparison to the pre‐launch period.

### 2.3 Impact of the program on monthly rates of referral to and use of Quitline for Aboriginal and Torres Strait Islander peoples: ITS analysis of monthly summarised data

Dataset 2 included the monthly count of Quitline data for Aboriginal and Torres Strait Islander peoples in CQ. In these analyses, the outcome variables were: (i) *referral* – the monthly count of referrals to Quitline received by the Health Contact Centre for Aboriginal and Torres Strait Islander clients including client self‐referrals and third‐party referrals; (ii) *participation* – the monthly count of initial Quitline counselling sessions delivered to Aboriginal and Torres Strait Islander clients; and (iii) *interaction* – the monthly count of total Quitline counselling sessions delivered to Aboriginal and Torres Strait Islander clients.

Data were available for the period between 1 January 2014 and 31 December 2019. We divided the whole period into three: period 1 (1 January 2014 to 31 January 2017) is the pre‐launch period of IQSP, a Quitline program comprised of telephone counselling sessions and 12‐weeks of free NRT (neither IQSP nor ‘10,000 Lives’ available during this period); period 2 (1 February 2017 to 31 August 2017) is the period between launching of IQSP by Quitline until ‘10,000 Lives’ commenced (only IQSP in operation); and period 3 (1 November 2017 to 31 December 2019) is the post‐launch period of ‘10,000 Lives’ (both IQSP and ‘10,000 Lives’ are in operation). We excluded a buffer period between 1 September 2017 and 31 October 2017 when ‘10,000 Lives’ started activities but had not officially launched.

We conducted an ITS analysis with this dataset to measure the change in the level of three outcomes: referrals to, participation in and interaction with Quitline in the post‐launch period of ‘10,000 Lives’ (period 3). The analysis also considered the impact of a co‐intervention (i.e. IQSP) that was launched by Queensland Quitline 7 months before the main intervention of interest (‘10,000 Lives’) was launched.

We followed the method outlined in Linden *et al*. [19] and Bernal *et al*. [20] and adapted the following equation to measure the impact by regressing an output series (*Y*) at time *t*:

$$Y_{t}=\beta_{0}+\beta_{1}*T+\beta_{2}*P_{t}+\beta_{3}*T*P_{t}+\beta_{4}*T*Q_{t}+\beta_{5}*T*Q_{t}+e_{t}$$

where *β*_0_ indicates the baseline level at *t* = 0, *β*_1_ is the outcome that indicates the time unit (monthly) increase (i.e. the underlying trend during the pre‐launch period of an intervention), $\beta_{2}$ is the level change following introduction of the co‐intervention (i.e. the IQSP); β_3_ indicates the trend change following IQSP commencement, β_4_ is the level change of outcome after launch of the main intervention of interest (‘10,000 Lives’), and β_5_ indicates the trend changes following the launch of ‘10,000 Lives’. We included the time variable (T), which is incremented monthly following the beginning of the study period, and two dummy variables: P_t_ (IQSP) and Q_t_ (‘10,000 Lives’) coded as 0 for pre‐intervention and 1 for post‐intervention. Finally, $e_{t}\;$ indicates the correlation in the error terms.

We performed the Poisson regression model, a generalised linear model that is generally used to model the count or rate data [21]. We checked for any autocorrelation in the models by plotting autocorrelation function and partial autocorrelation function, and seasonality by fitting Fourier terms (pairs of sine and cosine function) into our models [20]. We found no autocorrelation but adjusted the seasonal trends in the final models.

### 2.4 Participant involvement

We used routinely collected data from an anonymised individual level dataset and a summarised dataset for this impact evaluation.

### 2.5 Ethics statement

The study is approved by the Central Queensland Hospital and Health Service Human Research Ethics Committee; approval number: HREC/2019/QCQ/50602.

## 3 RESULTS

### 3.1 Impact on the number of individuals who were referred to Quitline by demography and Indigenous status

During the 26 months pre‐ and 26 months post‐launch of ‘10,000 Lives’, 4801 individuals were referred to Quitline (74.8% self‐referred and the remainder referred by a third party) for quit support. Of these referrals, 48.9% were female (4.7% unknown gender), 71.3% were aged between 30 and 64 years (mean age 42.5 ± 14.2) and 15.9% identified as Aboriginal and/or Torres Strait Islander (11.9% unknown Indigenous status). The number of referred individuals significantly increased in the post‐launch period among each demographic group. The overall increase in referrals was 101.2%. Increased referrals were relatively higher for females (105.1%), those aged between 14–17 years (169.7%) and 65+ years (122.3%), and Aboriginal and/or Torres Strait Islander peoples (205.6%). Most referrals (67.7%) were self‐referrals, but both self‐referrals and third‐party referrals increased by 114.4% and 76.8%, respectively, in the post‐launch period when compared to pre‐launch period. The increase of self‐referral was relatively higher among females (116.9%), those aged between 45–64 years (142.4%) and 65+ years (194.4%), and Aboriginal and Torres Strait Islander peoples (183.7%). The increase in third‐party referrals was relatively higher among females (87.3%), those aged between 14 and 44 years (92.4%), and Aboriginal and/or Torres Strait Islander peoples (231.7%). These results are shown in Tables S1, S2 and S3.

### 3.2 Impact on the number of individuals who participated in the Quitline program by demography and Indigenous status

Table 1 shows the demographic distribution of the individuals who participated in the Quitline program in the 52 months of pre‐ (26 months) and post‐launch (26 months) of ‘10,000 Lives’. In total, 3149 individuals participated in the Quitline program by completing an initial counselling session (assessment and quit planning support). Among the 3149 individuals, 48.3% were male (5% unknown gender), 48.3% were aged 45+ years and 13.4% identified as Aboriginal and/or Torres Strait Islander peoples (3.4% unknown Indigenous status). The mean age of the participants was 43.4 ± 13.7 (range 15–80 years). The overall increase in the number participating in the program post‐launch was 107.8% compared to the equivalent pre‐launch period. A statistically significantly higher increase was observed among females (125.6%), people aged 45+ years (137%) and Aboriginal and/or Torres Strait Islander peoples (212.7%).

**TABLE 1:** Comparative statistics of demographics and Indigenous status for individuals (*n* = 3149) from Central Queensland who participated (initial assessment call) in the Quitline program in the 26 months pre‐ and 26‐months post‐launch period of ‘10,000 lives’

Characteristics	Overall	Pre‐launch	Post‐launch	Relative increase in post‐launch period	*P*‐value*
No. of individual participated in Quitline program	3149	1023	2126	107.8	
Gender					<0.001
Female	1470 (46.7%)	446 (43.6%)	1024 (48.2%)	129.6	
Male	1522 (48.3%)	556 (54.3%)	966 (45.4%)	73.7	
Not stated	157 (5.0%)	21 (2.1%)	136 (6.4%)		
Age, years					0.006
Mean (SD)	43.4 (13.7)	42.4 (13.6)	43.9 (13.7)		
Range	15.0–80.0	15.0–79.0	15.0–80.0		
Age group, years					0.206
14–17	61 (1.9%)	21 (2.1%)	40 (1.9%)	90.5	
18–29	527 (16.7%)	189 (18.5%)	338 (15.9%)	78.8	
30–44	1047 (33.2%)	348 (34.0%)	699 (32.9%)	100.9	
45–64	1299 (41.3%)	404 (39.5%)	895 (42.1%)	121.	
65+	215 (6.8%)	61 (6.0%)	154 (7.2%)	152.5	
Indigenous status					<0.001
Neither Aboriginal nor Torres Strait Islander	2622 (83.3%)	861 (84.2%)	1761 (82.8%)	104.5	
Aboriginal and/or Torres Strait Islander peoples	421 (13.4%)	102 (10.0%)	319 (15.0%)	212.7	
Not stated	106 (3.4%)	60 (5.9%)	46 (2.2%)		

*Note*: Pre‐launch: 1 July 2015 to 31 August 2017 as 26 months pre‐launch period; post‐launch: 1 November 2017 to 31 December 2019 as 26 months post‐launch period.

* *P*‐value reflected as overall *P*‐value from chi‐square test result by comparing proportion (row percentage) between pre‐ and post‐launch group for categorical and analysis of variance for continuous variable.

### 3.3 Impact on the number of individuals who completed the Quitline program by demography and Indigenous status

Table 2 shows the demographics of Quitline program completers. During the 26 months pre‐ and 26 months post‐launch of ‘10,000 Lives’, 1149 individuals completed the program. Of them, 52.6% were male (4.4% unknown gender), 61.0% were aged 45+ years (mean age: 47.5 ± 13) and 13.4% identified as Aboriginal and/or Torres Strait Islander peoples (3.4% Indigenous status unknown). The number of individuals who completed the program substantially increased in the 26 months post‐launch period, with an overall increase of 330.7%; and even higher increases for females (484%), people aged 45+ years (368.7%) and Aboriginal and/or Torres Strait Islander peoples (350%).

**TABLE 2:** Comparative statistics (between 26 months pre‐ and 26 months post‐launch period) of demographics and Indigenous status of the individuals (*n* = 1194) who completed the fourth/final counselling session of Quitline program including the proportion using any quit method and who quit smoking in the subset who completed an evaluation call (*n* = 776)

Characteristics	Overall	Pre‐launch	Post‐launch	Relative increase in post‐launch	*P‐*value*
No. of individual completed the final/fourth counselling session	1194	225	969	330.7	
Gender					<0.001
Female	513 (43.0%)	75 (33.3%)	438 (45.2%)	484.0	
Male	628 (52.6%)	148 (65.8%)	480 (49.5%)	224.3	
Not stated	53 (4.4%)	2 (0.9%)	51 (5.3%)		
Age, years					0.039
Mean (SD)	47.5 (13.3)	45.8 (13.4)	47.9 (13.2)		
Range	15.0–80.0	15.0–79.0	15.0–80.0		
Age group, years					0.344
14–17	15 (1.3%)	3 (1.3%)	12 (1.2%)		
18–29	113 (9.5%)	28 (12.4%)	85 (8.8%)	203.6	
30–44	338 (28.3%)	66 (29.3%)	272 (28.1%)	312.1	
45–64	609 (51.0%)	111 (49.3%)	498 (51.4%)	348.6	
65+	119 (10.0%)	17 (7.6%)	102 (10.5%)	500.0	
Indigenous status					0.693
Neither Aboriginal nor Torres Strait Islander	1043 (87.4%)	196 (87.1%)	847 (87.4%)	332.1	
Aboriginal and/or Torres Strait Islander peoples	132 (11.1%)	24 (10.7%)	108 (11.1%)	350.0	
Not stated	19 (1.6%)	5 (2.2%)	14 (1.4%)		
Completed an evaluation call?					0.022
No	418 (35.0%)	64 (28.4%)	354 (36.5%)	453.1	
Yes	776 (65.0%)	161 (71.6%)	615 (63.5%)	282.0	
Used any quit assistance? (subset who completed an evaluation call: *n* = 776)					0.02
No	286 (36.9%)	72 (44.7%)	214 (34.8%)	197.2	
Yes	490 (63.1%)	89 (55.3%)	401 (65.2%)	350.6	
Quit smoking? (subset who completed an evaluation call: *n* = 776)					0.414
No	349 (45.0%)	77 (47.8%)	272 (44.2%)	253.2	
Yes	427 (55.0%)	84 (52.2%)	343 (55.8%)	308.3	

*Note*: Pre‐launch: 1 July 2015 to 31 August 2017 as 26 months pre‐launch period; post‐launch: 1 November 2017 to 31 December 2019 as post‐launch period (26 months).

* *P*‐value reflected as overall *P‐*value from chi‐square test result by comparing proportion (row percentage) between pre‐ and post‐launch group for categorical and analysis of variance for continuous variable.

### 3.4 Impact on the number of individuals who quit smoking after completion of Quitline program by demography and Indigenous status

Three months after the final counselling session, Quitline called these clients to record quit support use (e.g. NRT) and current smoking status. Of 776 individuals who completed an evaluation call in the period, 63.1% had used quit assistance and 55% reported smoking abstinence at the date of the evaluation call. The number of individuals who used any quit assistance increased by 350.6% in post‐launch when compared to pre‐launch period (Table 2). The increases were higher among females (517.9%) and people aged 45+ years (437.5%) (Table S4). The overall proportion of individuals who quit smoking (abstinent from smoking until evaluation call) after program completion was not significantly different (*P‐*value = 0.414) between pre‐ (52.2%) and post‐launch (58.8%) periods (Table 2). However, the total number who quit smoking substantially increased (308.3%) in the post‐launch period and the increase was relatively higher among females (425%), people aged 45+ years (376.1%) and Aboriginal and/or Torres Strait Islander peoples (342.9%) (Table 3).

**TABLE 3:** Comparative statistics of demographics of the individuals (*n* = 427) who quit smoking (as stated in the evaluation call at 3 months after the program completion) during 26 months pre‐ and 26 months post‐launch period of ‘10,000 lives’

Characteristics	Overall	Pre‐launch	Post‐launch	Relative increase in post‐launch	*p‐*value*
No. of individual quit smoking	427	84	343	308.3	
Gender					<0.001
Female	175 (41.0%)	28 (33.3%)	147 (42.9%)	425.0	
Male	239 (56.0%)	56 (66.7%)	183 (53.4%)	226.8	
Not stated	13 (3.0%)	0 (0.0%)	13 (3.8%)		
Age, years					0.006
Mean (SD)	48.5 (13.1)	45.5 (11.8)	49.2 (13.3)	8.1	
Range	16.0–80.0	19.0–73.0	16.0–80.0	−15.8	
Age group, years					0.206
14–17	4 (0.9%)	0 (0.0%)	4 (1.2%)		
18–29	24 (5.6%)	7 (8.3%)	17 (5.0%)	142.9	
30–44	134 (31.4%)	31 (36.9%)	103 (30.0%)	232.3	
45–64	212 (49.6%)	42 (50.0%)	170 (49.6%)	304.8	
65+	53 (12.4%)	4 (4.8%)	49 (14.3%)	1125.0	
Indigenous status					<0.001
Neither Aboriginal nor Torres Strait Islander	384 (89.9%)	76 (90.5%)	308 (89.8%)	305.3	
Aboriginal and/or Torres Strait Islander peoples	38 (8.9%)	7 (8.3%)	31 (9.0%)	342.9	
Not stated	5 (1.2%)	1 (1.2%)	4 (1.2%)		

*Note*: Pre‐launch: 1 July 2015 to 31 August 2017 as 26 months pre‐launch period; post‐launch: 1 November 2017 to 31 December 2019 as post‐launch period (26 months).

* *P*‐value reflected as overall *P*‐value from chi‐square test result by comparing proportion (row percentage) between pre‐ and post‐launch group for categorical and analysis of variance for continuous variable.

### 3.5 Validation of the impact for Aboriginal and Torres Strait Islander peoples: Results from ITS analysis

This ITS analysis indicates an overall increasing trend in monthly referral to, participation in and interaction with Quitline for Aboriginal and Torres Strait Islander peoples in CQ during 2014 to 2019. All outcomes assessed showed moderate improvements after the introduction of the 12‐weeks‐free NRT in February 2017 (Period 2); representing increases of 48.1% (95% confidence interval [CI] 14.1–92.4%) for referrals to Quitline, 90.3% (95% CI 22.2–196.4%) for participation in Quitline and 52.6% (95% CI 21.0–92.4%) for interaction with Quitline. However, all outcomes significantly increased (*P‐*value <0.001) in the post‐launch period, representing 299.9% (95% CI 117.9–634.1%), 457.2% (95% CI 108.4–1389.8%) and 469.8% (95% CI 241.0–851.9%) increase for referrals to, participation in and interaction with Quitline, respectively. Table 4 demonstrates the results of a single ITS analysis, including adjustment for seasonal effects, which result in substantial increases in the outcomes observed in the post‐launch period. The model plot of the ITS analysis is shown in Figure S5.

**TABLE 4:** Results from a single interrupted time series analysis of monthly referrals to, participation in, and interaction with Quitline by aboriginal and Torres Strait islander peoples in Central Queensland in the study period between January 2014 and December 2019

Predictors	Referral		Participation		Interaction	
	IRR [95% CI]	Seasonality adjusted IRR [95% CI]	IRR [95% CI]	Seasonality adjusted IRR [95% CI]	IRR [95% CI]	Seasonality adjusted IRR [95% CI]
Baseline trend (slope)	1.023***	1.027***	1.008	1.009	1.017***	1.018***
	[1.014, 1.033]	[1.018, 1.037]	[0.994, 1.023]	[0.994, 1.024]	[1.009, 1.025]	[1.010, 1.026]
Level in period 2	1.481**	1.223	1.903**	1.795*	1.526***	1.415**
	[1.141, 1.924]	[0.936, 1.599]	[1.222, 2.964]	[1.136, 2.836]	[1.210, 1.924]	[1.115, 1.797]
Level in period 3	3.999***	4.415***	5.572***	6.903***	5.698***	6.797***
	[2.179, 7.341]	[2.365, 8.242]	[2.084, 14.898]	[2.514, 18.951]	[3.410, 9.519]	[4.017, 11.501]
Slope in period 3	0.982**	0.981**	0.980*	0.978*	0.980***	0.978***
	[0.970, 0.993]	[0.969, 0.993]	[0.962, 1.000]	[0.959, 0.997]	[0.970, 0.990]	[0.968, 0.988]
*Model check*						
AIC	*572.589*	*537.64*	*365.190*	*362.660*	*506.293*	*490.493*
BIC	*583.831*	*553.379*	*376.432*	*378.400*	*517.536*	*506.233*
*R* ^*2*^	*0.677*	*0.767*	*0.704*	*0.744*	*0.928*	*0.901*

*Note*: Referral: the number of Aboriginal and/or Torres Strait Islander client referrals to the Quitline service received by the Health Contact Centre of Queensland Health. This could be either by client self‐referral or third‐party referral from another person or organisation. Participation/Initial counselling session: the number of Aboriginal and Torres Strait Islander clients who completed at least the first Quitline call. Interaction/total counselling sessions: the number of individual Quitline telephone counselling sessions completed in the study area comprising of initial and subsequent calls. Period 1 = Pre‐launch period of any intervention (January 2014 to January 2017), *N* = 44 months; Period 2 = Time between launching of Intensive Quit Support Program by Quitline until the ‘10,000 Lives’ started to work (February 2017 to August 2017), *N* = 07 months; Period 3 = post‐launch period of ‘10,000 Lives’ (November 2017 to December 2019), *N* = 26 months; ^@^Data of this outcome during pre‐intervention is only for 7 periods which may cause error in estimation and adjustment of seasonality is not viable.

AIC, Akaike information criteria; BIC, Bayesian information criterion; CI, confidence interval; IRR, incidence rate ratio.

*** P < 0.001;

** *P* < 0.01;

* *P* < 0.05.

## 4 DISCUSSION

The current study investigated the impact of ‘10,000 Lives’ on referrals to, uptake and outcomes of Quitline services by different demographic characteristics and for Aboriginal and Torres Strait Islander peoples. The ‘10,000 Lives’ initiative was effective in increasing the number of different outcomes: referrals to and use of Quitline services, and smoking cessation among different population groups, including all age and gender groups, and non‐Indigenous and Aboriginal and Torres Strait Islander peoples. While the outcomes increased substantially for both males and females in post‐launch of the ‘10,000 Lives’, the rate of increase was relatively higher among females. Although females (11.9%) have substantially lower smoking prevalence than males (18.9%) in CQ, the use of Quitline followed by them reporting smoking abstinence was found relatively higher [5]. This result is consistent with the population level analysis of nationwide historical (1998–2017) data for Australia [22]. The analysis showed that females made significantly more calls to Quitline and more attempts to quit smoking compared to males [22]. Importantly, the initiative boosted the referrals and use of Quitline for Aboriginal and Torres Strait Islander peoples. More than four‐fold increases were observed in the number of individuals aged between 45 and 64 years who completed the Quitline program by attending the four quit support sessions during the post‐launch period. More than three‐fold increases were observed in the number of Aboriginal and Torres Strait Islander peoples who were referred to, participated in the initial counselling session for assessment and plan for quit support counselling sessions, and completed the counselling sessions. Overall, ‘10,000 Lives’ increased the use of Quitline among populations where smoking prevalence is higher, including the 45–64 years age group and Aboriginal and Torres Strait Islander peoples. However, more attention needs to be given to engaging men in smoking cessation support services such as Quitline because they are more likely to smoke than women, but also use Quitline less frequently.

Only a third of Quitline referrals were from third parties, while the majority of clients were self‐referred. This indicates that promotion of Quitline had a good reach directly to the smoking population in CQ. The substantial increase of both self‐ and third‐party referrals in all population groups after the ‘10,000 Lives’ launch indicates that all Quitline pathways were significantly boosted by the program.

The initiative was very effective for the Aboriginal and Torres Strait Islander population in CQ, where smoking prevalence is more than double that of non‐Indigenous people [8]. While the increased of use of any quit assistance (e.g. NRT) during the post‐launch period was higher for non‐Indigenous people, the increase in successful smoking cessation (abstinent from smoking for at least 3 months after completing the program) was relatively higher among Aboriginal and Torres Strait Islander Quitline clients. We validated this result for several specific outcome measures for the Aboriginal and Torres Strait Islander population by ITS analysis. We found a sharp rise in the level of monthly referrals to, participation in the initial session and total counselling sessions with Quitline immediately after launching ‘10,000 Lives’ in November 2017. However, the monthly numbers of referrals to and participation in the counselling sessions were further boosted in the middle of the year and dropped at the end of each year. The decrease in the number of referrals to, and participation in the initial counselling session and total counselling sessions of Quitline during the end of year and following new year may be due to the long summer holidays and festive period. However, contrary findings such as increased use of Quitline during December–January, which could be explained by New Year's resolutions and clean indoor air restrictions due to cold months, were reported in the northern hemisphere (America and Europe) [23].

Studies conducted in other countries have reported similar findings to our study. Although significant disparities exist in uptake of Quitline in different age, gender, socio‐economic and ethnic groups [24, 25], initiatives such as tailoring Quitline services, incentivising people who smoke and referrers to use Quitline services can help to effectively address disparities in Quitline use [26, 27]. Our study produced evidence that an initiative like ‘10,000 Lives’ can effectively increase the uptake of those programs and reduce the disparities in different population groups.In Australia, referrals to and uptake of Quitline among Aboriginal and Torres Strait Islander peoples are lower than non‐Indigenous people. Finding culturally appropriate ways to improve access to smoking cessation support is crucial to increase equitable provision of health services [28, 29]. Australian Quitlines offer counselling by Aboriginal and Torres Strait Islander counsellors to provide culturally safe smoking cessation assistance. Health professionals working with Aboriginal and Torres Strait Islander peoples can play an important role in referring them to Quitline. But referrers need to be trained, upskilled and confident with cultural appropriateness in order to provide effective support to their Indigenous clients [30]. Queensland Quitline's initiative for priority populations, Queensland Health's Quality Improvement Payment program and targeted smoking cessation programs (i.e. ‘Quit for You…Quit for Baby’, ‘Quit for You’, ‘Yarn to Quit’, B.strong) have contributed to an increase in the uptake of Quitline services among the Aboriginal and Torres Strait Islander population [10]. Our study further demonstrates that initiatives such as ‘10,000 Lives’ can add additional value to these valuable interventions for priority populations by promoting the service among the clinical and community champions who can contribute by referring clients or colleagues to Quitline. Additionally, collaboration with other programs and initiatives that work to reduce the prevalence of smoking in priority populations can support further uptake of Quitline services [16].

### 4.1 Study limitations

Since only secondary data were available for this analysis, the current evaluation was limited in similar ways to all studies using administrative data not originally collected for research purposes. These include challenges associated with data accuracy and completeness, and the restricted format of the available data. For example, only 25% (*n* = 776) of Quitline clients completed the final evaluation call that determined the Quitline program's outcome (current smoking status). Again, we cannot unequivocally state that the observed impact was solely a result of ‘10,000 Lives’ since many other initiatives were run at the same time in this region. However, a comprehensive approach to our analysis, including the ITS analysis, supports the contribution of ‘10,000 Lives’ to these outcomes. Including a control group (comparison area) in this analysis would further validate our findings, but we did not have denominator population (number of Aboriginal and Torres Strait Islander people who smoke per geographic area over the period) data consistently available for the intervention area and a comparable control area.

## 5 CONCLUSIONS

A locally coordinated initiative that mobilised clinical and community champions to promote smoking cessation, substantially enhanced the uptake of, referrals to, and use of the Quitline service and increased smoking cessation among women, the 45–64 years age group and Aboriginal and Torres Strait Islander peoples. However, further attention should be given to men because they have a higher prevalence but lower use of Quitline. The ‘10,000 Lives’ initiative can be seen as key in supporting future programs to address the inequities in referrals to and use of Quitline. The comprehensive approach of the initiative can be replicated in other areas where smoking cessation interventions like Quitline are available, but uptake is suboptimal.

## AUTHOR CONTRIBUTIONS

Arifuzzaman Khan, Sheleigh Lawler and Coral Gartner conceived and designed the study. Arifuzzaman Khan communicated and collected data from the Health Contact Centre of Queensland Health. Arifuzzaman Khan performed the primary analysis and interpretation of the data. Arifuzzaman Khan, Kalie Green, Linda Medlin, Gulam Khandaker, Sheleigh Lawler and Coral Gartner critically reviewed the data analysis and interpretation. Linda Medlin critically reviewed the manuscript from the perspective of cultural appropriateness. Arifuzzaman Khan drafted the manuscript, and all authors contributed with critical revisions to the manuscript's content. All authors approved the final version of the manuscript.

## FUNDING INFORMATION

The research is funded by the collaborative research grant between School of Public Health at the University of Queensland and Central Queensland Public Health Unit, which is awarded by the Central Queensland Hospital and Health Service (CQHHS93907). The lead author (AK) is supported by a University of Queensland Research Training Scholarship and a Research Higher Degree Top‐up Scholarship.

## CONFLICT OF INTEREST

The authors declare no conflict of interest.

## Supporting information


**Table S1** Comparative statistics of demographics and the source of referrals for individuals (*n* = 4801) referred to Quitline from Central Queensland in 26 months pre‐ and 26 months post‐launch period of ‘10,000 Lives’.
**Table S2**. Comparative statistics of demographics of individuals (*n* = 3248) who self‐referred to Quitline in the 26‐months‐pre‐ and 26‐months‐post‐launch period of ‘10,000 Lives’.
**Table S3**. Comparative statistics of demographics of individuals (*n* = 1553) referred to Quitline by any third‐party in 26 months pre‐ and 26 months post‐launch period of ‘10,000 Lives’.
**Table S4**. Comparative statistics of demographics of the individuals (*n* = 490) who used any quit assistance (as stated in the evaluation call at 3 months after the program completion) during 26 months pre‐ and 26 months post‐launch period of ‘10,000 Lives’.
**Figure S1**. Model plots from single interrupted time series analysis showing the monthly count of (i) referral to; (ii) participation; and (iii) interaction with Quitline by Aboriginal and/or Torres Strait Islander peoples in Central Queensland over the study period between January 2014 to December 2019.
