# Supplementary material for: Impact of the ‘10,000 lives’ program on Quitline referrals, use and outcomes by demography and Indigenous status
Source: Drug Alcohol Rev. 2022 Jul 13;41(7):1499–509. doi: 10.1111/dar.13499 (PMC9796440; doi:10.1111/dar.13499)
Supplement: Supplementary file 1 — Table S1 Comparative statistics of demographics and the source of referrals for individuals (n = 4801) referred to Quitline from Central Queensland in 26 months pre‐ and 26 months post‐launch period of ‘10,000 Lives’. Table S2. Comparative statistics of demographics of individuals (n = 3248) who self‐referred to Quitline in the 26‐months‐pre‐ and 26‐months‐post‐launch period of ‘10,000 Lives’. Table S3. Comparative statistics of demographics of individuals (n = 1553) referred to Quitline by any third‐party in 26 months pre‐ and 26 months post‐launch period of ‘10,000 Lives’. Table S4. Comparative statistics of demographics of the individuals (n = 490) who used any quit assistance (as stated in the evaluation call at 3 months after the program completion) during 26 months pre‐ and 26 months post‐launch period of ‘10,000 Lives’. Figure S1. Model plots from single interrupted time series analysis showing the monthly count of (i) referral to; (ii) participation; and (iii) interaction with Quitline by Aboriginal and/or Torres Strait Islander peoples in Central Queensland over the study period between January 2014 to December 2019. [file DAR-41-1499-s001.docx]

**Table S1. Comparative statistics of demographics and the source of referrals for individuals (n=4801) referred to Quitline from Central Queensland in 26 months pre- and 26 months post-launch period of “10,000 Lives”**

| **Characteristics** | **Overall** | **Pre-launch** | **Post-launch** | ***Relative increase in post-launch*** | ***P-*value*** |
| --- | --- | --- | --- | --- | --- |
| **No. of individual referred** | **4801** | **1594** | **3207** | **101.2%** |  |
| *Gender* |  |  |  |  | **< 0.001** |
| **Female** | 2346 (48.9%) | 769 (48.2%) | **1577 (49.2%)** | **105.1%** |  |
| Male | 2230 (46.4%) | 802 (50.3%) | 1428 (44.5%) | 78.1% |  |
| *Not stated* | *225 (4.7%)* | *23 (1.4%)* | *202 (6.3%)* |  |  |
| *Age, years* |  |  |  |  | **0.036** |
| - Mean (SD) | 42.5 (14.2) | 41.9 (14.0) | 42.8 (14.3) |  |  |
| - Range | 15.0-84.0 | 15.0-84.0 | 15.0-80.0 |  |  |
| *Age group, years* |  |  |  |  | 0.061 |
| **14-17** | 122 (2.5%) | 33 (2.1%) | **89 (2.8%)** | **169.7%** |  |
| 18-29 | 927 (19.3%) | 335 (21.0%) | 592 (18.5%) | 76.7% |  |
| 30-44 | 1583 (33.0%) | 541 (33.9%) | 1042 (32.5%) | 92.6% |  |
| 45-64 | 1837 (38.3%) | 582 (36.5%) | 1255 (39.1%) | 115.6% |  |
| **65+** | 332 (6.9%) | 103 (6.5%) | **229 (7.1%)** | **122.3%** |  |
| *Indigenous status* |  |  |  |  | **< 0.001** |
| Neither Aboriginal nor Torres Strait Islander | 3502 (72.9%) | 1222 (76.7%) | 2280 (71.1%) | 86.6% |  |
| **Aboriginal and/or Torres Strait Islander peoples** | 730 (15.2%) | 180 (11.3%) | 550 (17.1%) | **205.6%** |  |
| *Not stated* | *569 (11.9%)* | *192 (12.0%)* | *377 (11.8%)* |  |  |
| *Source of referrals* |  |  |  |  | **0.003** |
| **Self** | 3248 (67.7%) | 1033 (64.8%) | 2215 (69.1%) | **114.4%** |  |
| Third-party** | 1553 (32.3%) | 561 (35.2%) | 992 (30.9%) | 76.8% |  |

Pre-launch: 1 July 2015 to 31 August 2017 as 26 months pre-launch period; Post-launch: 1 November 2017 to 31 December 2019 as 26 months post-launch period. **P*-value reflected as overall *P*-value from chi-square test result by comparing proportion (row percentage) between pre- and post-launch group for categorical and analysis of variance for continuous variable. ** Vast majority (>99%) of all third-party referrals were from health professionals in all time periods.

**Table S2. Comparative statistics of demographics of individuals (n=3248) who self-referred to Quitline in the 26-months-pre- and 26-months-post-launch period of “10,000 Lives”**

| **Characteristics** | **Overall** | **Pre-launch** | **Post-launch** | ***Relative increase in post-launch*** | ***P-value**** |
| --- | --- | --- | --- | --- | --- |
| **No. of individual self-referred to Quitline** | **3248** | **1033** | **2215** | **114.4%** |  |
| *Gender* |  |  |  |  | **< 0.001** |
| **Female** | 1461 (45.0%) | 461 (44.6%) | **1000 (45.1%)** | **116.9%** |  |
| Male | 1575 (48.5%) | 552 (53.4%) | 1023 (46.2%) | 85.3% |  |
| *Not stated* | *212 (6.5%)* | *20 (1.9%)* | *192 (8.7%)* |  |  |
| *Age, years* |  |  |  |  | **< 0.001** |
| Mean (SD) | 43.6 (13.6) | 42.1 (13.3) | 44.3 (13.7) |  |  |
| Range | 15.0 - 84.0 | 16.0 - 84.0 | 15.0 - 80.0 |  |  |
| *Age group, years* |  |  |  |  | **0.002** |
| 14-17 | 68 (2.1%) | 23 (2.2%) | 45 (2.0%) | 95.7% |  |
| 18-29 | 510 (15.7%) | 189 (18.3%) | 321 (14.5%) | 69.8% |  |
| 30-44 | 1095 (33.7%) | 369 (35.7%) | 726 (32.8%) | 96.7% |  |
| **45-64** | 1366 (42.1%) | 399 (38.6%) | **967 (43.7%)** | **142.4%** |  |
| **65+** | 209 (6.4%) | 53 (5.1%) | **156 (7.0%)** | **194.3%** |  |
| *Indigenous status* |  |  |  |  | **0.029** |
| Neither Aboriginal nor Torres Strait Islander | 2737 (84.3%) | 887 (85.9%) | 1850 (83.5%) | 108.6% |  |
| **Aboriginal and/or Torres Strait Islander peoples** | 376 (11.6%) | 98 (9.5%) | **278 (12.6%)** | **183.7%** |  |
| *Not stated* | *135 (4.2%)* | *48 (4.6%)* | *87 (3.9%)* | *81.3%* |  |

Pre-launch: 1 July 2015 to 31 August 2017 as 26 months pre-launch period; Post-launch: 1 November 2017 to 31 December 2019 as post-launch period (26 months). **P*-value reflected as overall *P*-value from chi-square test result by comparing proportion (row percentage) between pre- and post-launch group for categorical and analysis of variance for continuous variable.

**Table S3. Comparative statistics of demographics of individuals (n=1553) referred to Quitline by any third-party in 26 months pre- and 26 months post-launch period of “10,000 Lives”**

| **Characteristics** | **Overall** | **Pre-launch** | **Post-launch** | ***Relative increase in post-launch*** | ***P-*value*** |
| --- | --- | --- | --- | --- | --- |
| **No. of individual referred to Quitline by a third-party** | **1553** | **561** | **992** | **76.8%** |  |
| *Gender* |  |  |  |  | 0.245 |
| **Female** | 885 (57.0%) | 308 (54.9%) | **577 (58.2%)** | **87.3%** |  |
| Male | 655 (42.2%) | 250 (44.6%) | 405 (40.8%) | 62.0% |  |
| *Not stated* | *13 (0.8%)* | *3 (0.5%)* | *10 (1.0%)* |  |  |
| *Age, years* |  |  |  |  | **0.007** |
| Mean (SD) | 40.1 (15.2) | 41.5 (15.2) | 39.3 (15.1) |  |  |
| Range | 15.0 - 81.0 | 15.0 - 81.0 | 15.0 - 80.0 |  |  |
| *Age group, years* |  |  |  |  | **0.036** |
| **14-17** | 54 (3.5%) | 10 (1.8%) | **44 (4.4%)** | **340.0%** |  |
| 18-29 | 417 (26.9%) | 146 (26.0%) | **271 (27.3%)** | **85.6%** |  |
| 30-44 | 488 (31.4%) | 172 (30.7%) | **316 (31.9%)** | **83.7%** |  |
| **45-64** | 471 (30.3%) | 183 (32.6%) | 288 (29.0%) | 57.4% |  |
| **65+** | 123 (7.9%) | 50 (8.9%) | 73 (7.4%) | 46.0% |  |
| *Indigenous status* |  |  |  |  | **< 0.001** |
| Neither Aboriginal nor Torres Strait Islander | 765 (49.3%) | 335 (59.7%) | 430 (43.3%) | 28.4% |  |
| **Aboriginal and/or Torres Strait Islander peoples** | 354 (22.8%) | 82 (14.6%) | **272 (27.4%)** | **231.7%** |  |
| *Not stated* | *434 (27.9%)* | *144 (25.7%)* | *290 (29.2%)* | *101.4%* |  |

Pre-launch: 1 July 2015 to 31 August 2017 as 26 months pre-launch period; Post-launch: 1 November 2017 to 31 December 2019 as post-launch period (26 months). **P*-value reflected as overall *P*-value from chi-square test result by comparing proportion (row percentage) between pre- and post-launch group for categorical and analysis of variance for continuous variable.

**Table S4. Comparative statistics of demographics of the individuals (n=490) who used any quit assistance (as stated in the evaluation call at 3 months after the program completion) during 26 months pre- and 26 months post-launch period of “10,000 Lives”**

| **Characteristics** | **Overall** | **Pre-launch** | **Post-launch** | ***Relative increase in post-launch*** | ***P-*value*** |
| --- | --- | --- | --- | --- | --- |
| **No. of individuals who used any quit assistance (e.g. NRT)** | **490** | **89** | **401** | **350.6%** |  |
| *Gender* |  |  |  |  | **<0.001** |
| **Female** | 201 (100.0%) | 28 (13.9%) | **173 (86.1%)** | **517.9%** |  |
| Male | 267 (100.0%) | 61 (22.8%) | 206 (77.2%) | 237.7% |  |
| *Not stated* | *22 (100.0%)* | *0 (0.0%)* | *22 (100.0%)* |  |  |
| *Age, years* |  |  |  |  | **0.006** |
| Mean (SD) | 48.5 (12.9) | 45.9 (12.3) | 49.1 (12.9) | 7.0% |  |
| Range | 16.0 - 80.0 | 18.0 - 79.0 | 16.0 - 80.0 | -11.1% |  |
| *Age group, year* |  |  |  |  | 0.206 |
| 14-17 | 6 (100.0%) | 1 (16.7%) | 5 (83.3%) | 400.0% |  |
| 18-29 | 27 (100.0%) | 6 (22.2%) | 21 (77.8%) | 250.0% |  |
| 30-44 | 151 (100.0%) | 34 (22.5%) | 117 (77.5%) | 244.1% |  |
| **45-64** | 253 (100.0%) | 42 (16.6%) | **211 (83.4%)** | **402.4%** |  |
| **65+** | 53 (100.0%) | 6 (11.3%) | **47 (88.7%)** | **683.3%** |  |
| *Indigenous status* |  |  |  |  | **<0.001** |
| Neither Aboriginal nor Torres Strait Islander | 438 (100.0%) | 79 (18.0%) | 359 (82.0%) | 354.4% |  |
| **Aboriginal and/or Torres Strait Islander peoples** | 45 (100.0%) | 9 (20.0%) | **36 (80.0%)** | **300.0%** |  |
| *Not stated* | *7 (100.0%)* | *1 (14.3%)* | *6 (85.7%)* |  |  |

Pre-launch: 1 July 2015 to 31 August 2017 as 26 months pre-launch period, Post-launch: 1 November 2017 to 31 December 2019 as post-launch period (26 months). **P*-value reflected as overall *P*-value from chi-square test result by comparing proportion (row percentage) between pre- and post-launch group for categorical and analysis of variance for continuous variable.

**Figure S1. Model plots from single interrupted time series analysis showing the monthly count of (i) referral to; (ii) participation; and (iii) interaction with Quitline by Aboriginal and/or Torres Strait Islander peoples in Central Queensland over the study period between January 2014 to December 2019.**


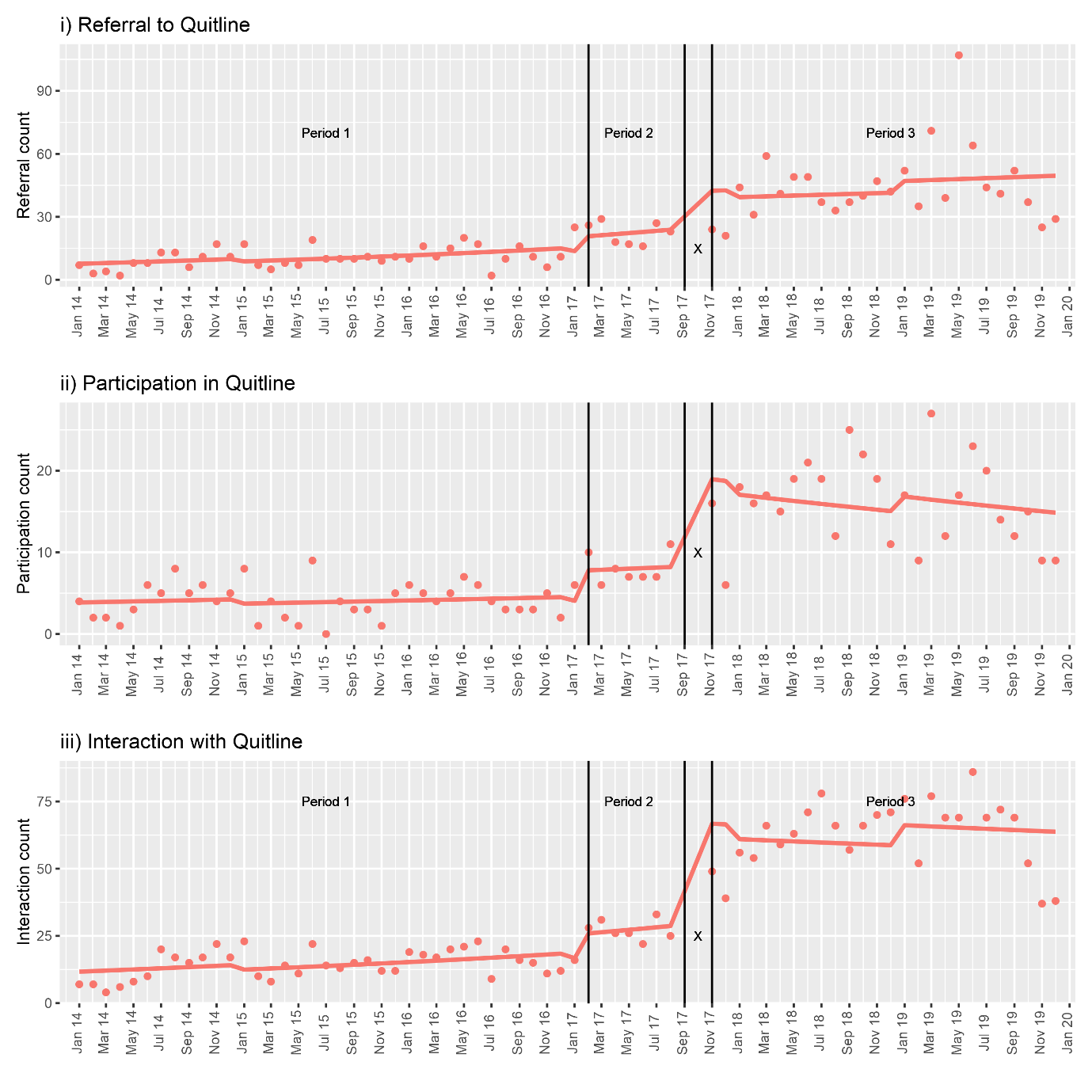


Referral: the number of client referrals to the Quitline service received by the Health Contact Centre of Queensland Health. This could be either by client self-referral or third-party referral from another person or organisation. Participation (initial counselling session): the number of clients who completed at least the first Quitline call. Interaction (total counselling session): the number of individual Quitline telephone counselling sessions (support calls) completed in the study area comprising of initial and subsequent calls. Period 1: pre-launch period of any intervention (1 January 2014 to 31 January 2017); Period 2: time between launching of the Intensive Quit Support Program by Quitline until the “10,000 Lives” started to work (1 February 2017 to 31 August 2017); Period 3: post-launch period of “10,000 Lives” (1 November 2017 to 31 December 2019); X: buffer period (1 September 2017 to 31 October 2017) which is excluded from analysis.
